# Correlates of perceived stress among community-dwelling older African Americans

**DOI:** 10.1371/journal.pone.0260749

**Published:** 2021-12-01

**Authors:** Crystal M. Glover, Ana W. Capuano, Robert S. Wilson, David A. Bennett, Lisa L. Barnes

**Affiliations:** 1 Rush Alzheimer’s Disease Center, Rush University Medical Center, Chicago, Illinois, United States of America; 2 Department of Psychiatry and Behavioral Sciences, Rush Medical College, Chicago, Illinois, United States of America; 3 Department of Neurological Sciences, Rush Medical College, Chicago, Illinois, United States of America; Indiana University Purdue University at Indianapolis, UNITED STATES

## Abstract

**Background:**

The purpose of this study was to identify correlates of perceived stress among older African Americans.

**Methods and findings:**

Guided by the National Institute on Aging’s (NIA) Health Disparities Research Framework, we grouped correlates into four levels–environmental, sociocultural, behavioral, and biological, and performed a cross-sectional analysis using ordinal logistic regression models. Participants included 722 African Americans [mean age = 73.61 years (SD = 6.33)] from the Minority Aging Research Study (MARS). Several protective correlates from environmental (e.g., larger life space), sociocultural (e.g., larger social network size), behavioral (e.g., more purpose in life), and biological (e.g., higher global cognition) levels were associated with a lower odds of having higher levels of perceived stress.

**Conclusions:**

Perceived stress was associated with established and novel correlates from every level. Future research is needed to examine how changes in these correlates may impact perceived stress in older African Americans.

## Introduction

Older adults (age 65 years and older) will comprise more than 20% of the United States population by 2050, an increase from nearly 14% in 2012 [1]. This population will also become increasingly diverse as nearly 40% of all older adults will belong to racial and ethnic minoritized groups by 2050, up from barely 20% in 2012 [1]. Hence, a larger and more diverse group of Americans may face unique challenges presented by aging such as changes to income and financial burden [2], decisions regarding housing and living arrangements [3], and changes in social networks and social activities [4]. Older adults who are racial minorities [5] may be at higher risk of experiencing these challenges as they are more likely to occupy multiple minoritized statuses, such as being African American, having advanced age, and lower income; and to encounter negative experiences such as discrimination associated with these statuses [6]. These age- and race-related challenges can induce feelings or perceptions of stress.

Perceived stress denotes a person’s subjective appraisal of stress in his or her life and whether s/he has the resources (e.g., monetary or positive lifestyle behaviors such as physical activity) to cope with the stress [7, 8]. It is plausible that differential levels of perceived stress may impact or mitigate the health outcomes of older adults [9]. As such, previous studies have largely focused on perceived stress as a predictor of various health outcomes, with few studies examining potential antecedents of perceived stress [10, 11]. Given the association of perceived stress with adverse health outcomes, it is important to reduce a person’s exposure to negative factors that can impact their perceptions of stress. Hence, we must identify antecedents of perceived stress as intervention targets for mitigating, addressing, and coping with higher levels of perceived stress among older adults, especially those belonging to racial and ethnic minoritized groups. Overall, loneliness, financial strain, neighborhood stress (e.g., feeling less safe), age discrimination, and younger age are associated with higher levels of perceived stress among either predominately White adults or men across the life span [12, 13]. Although perceived stress is undoubtedly pervasive for all older adults, it is particularly critical to understand antecedents of perceived stress among older African Americans–a group of people that tends to have well-documented and objective stressful life experiences–and for whom little is known about this topic [14, 15].

The purpose of the current study was to identify correlates of perceived stress among older African Americans. The National Institute on Aging’s (NIA) Health Disparities Research Framework provided an *a priori* conceptual structure to group the many factors available in the current study [16]. We used this framework [16] to guide our study because it includes a wide range of factors within different levels of analysis that have been found to be important across the life course through decades of health disparities research among demographically diverse aging populations, including older African Americans. The NIA Health Disparities Research Framework is meant to guide future research on health disparities in aging and considers four interrelated levels of analysis: 1) environmental–representing domains associated with a person’s physical and social settings that pertain to geography (e.g., neighborhood), political contexts, socioeconomic conditions, and health care; 2) sociocultural–representing domains associated with group-based cultural, social, and related psychological phenomena; 3) behavioral–representing domains associated with individual behaviors and psychological processes; and 4) biological–representing domains associated with physiological, genetic, and cellular processes [16]. The framework also highlights fundamental factors (e.g., race, ethnicity, socioeconomic status, disability status, and sex and gender identities) that determine priority populations for health disparities research and that should be considered for all levels of analysis [16]. According to the NIA Health Disparities Research Framework, older African Americans represent a priority population or one at risk for health disparities in aging.

**Fig 1:**
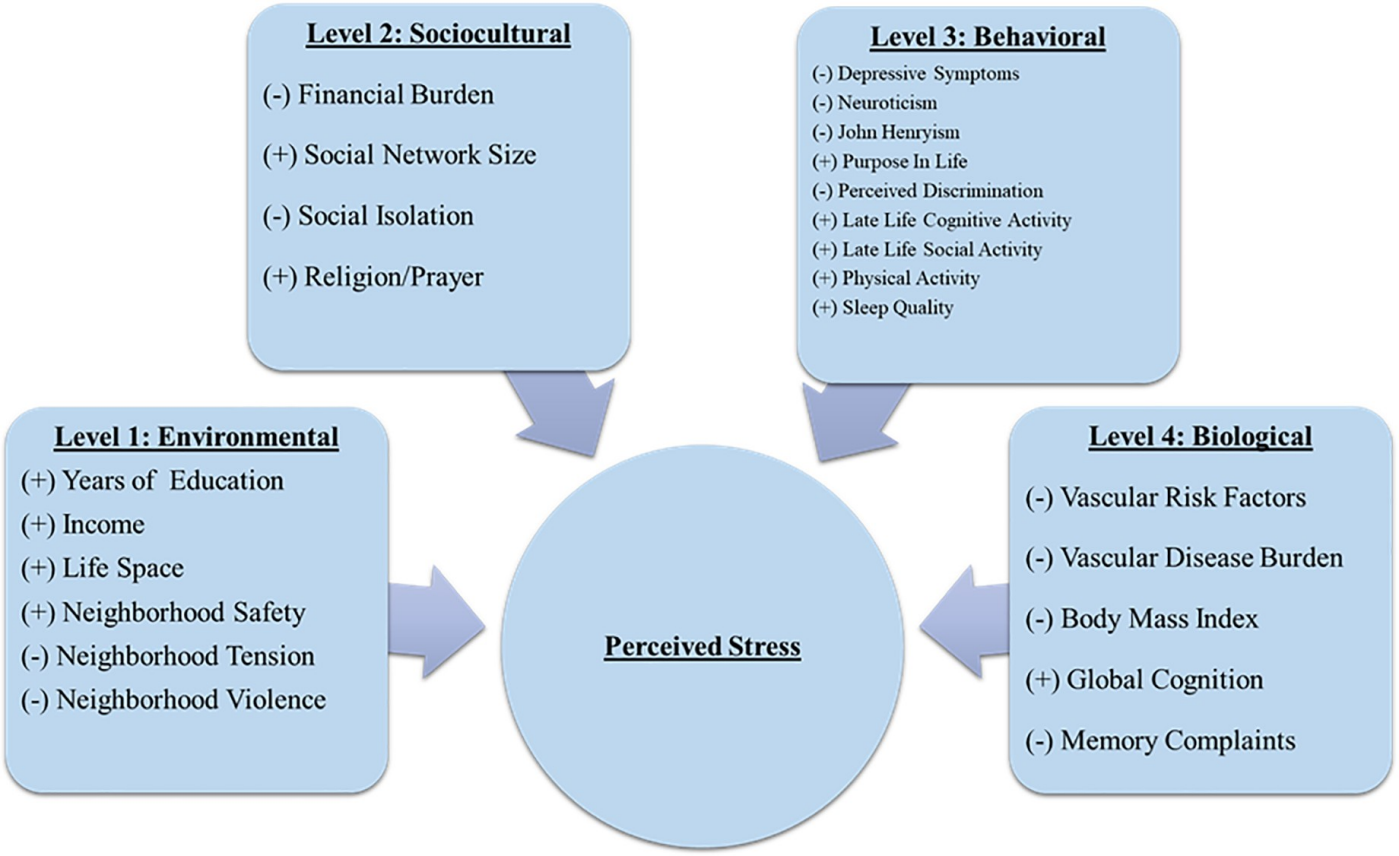
Hypothesized relationships between correlates* within each level and perceived stress. *(+) Indicates a positive correlate that we hypothesize as associated with lower levels of perceived stress. *(-) Indicates a negative correlate that we hypothesize as associated with higher levels of perceived stress.

The current study did not aim to test the validity of the NIA Health Disparities Research Framework; rather, we used the framework as an organizing structure to determine whether correlates at each level were more or less important in understanding perceptions of stress among older African Americans. See Table 1. As such, we hypothesized that more positive-oriented or protective correlates within each level of analysis—environmental (i.e., more years of education, higher income, larger life space, and greater neighborhood safety), sociocultural (i.e., larger social network size and higher frequency of prayer), behavioral (i.e., more purpose in life; more late life cognitive, social, and physical activities; and better sleep quality), and biological (i.e., higher cognition)—would be associated with lower levels of perceived stress among older African Americans. Conversely, we hypothesized that correlates deemed more negative or risk factors within each level of analysis—environmental (i.e., more neighborhood tension and violence), sociocultural (i.e., more financial burden and more social isolation), behavioral (i.e., more depressive symptoms, higher neuroticism, higher John Henryism, and higher levels of self-reported discrimination), and biological (i.e., more vascular risk factors and vascular disease burden, and more memory complaints)—would be associated with higher levels of perceived stress. Finally, we also included marital status and body mass index (BMI) as potential correlates of perceived stress in older African Americans and included them in the sociocultural and biological levels, respectively. We hypothesized that being married would be associated with lower levels of perceived stress while having a higher BMI would be associated with higher levels of perceived stress. See Fig 1.

**Table 1:** Potential correlates of perceived stress among older African Americans by level of analysis*.

Levels of Analysis				
**Correlates**	**Environmental**	**Sociocultural**	**Behavioral**	**Biological**
	• Years of Education • Income • Life Space • Neighborhood Safety • Neighborhood Tension • Neighborhood Violence	• Financial Burden • Marital Status • Social Network Size • Social Isolation • Religion/ Frequency of Prayer	• Depressive Symptoms • Neuroticism • John Henryism • Purpose in Life • Perceived Discrimination • Late Life Cognitive Activity • Late Life Social Activity • Physical Activity • Sleep Quality	• Vascular Risk Factors • Vascular Disease Burden • Body Mass Index • Global Cognition • Memory Complaints
**Fundamental Factors**				
Gender and Age				

*Adapted from the National Institute on Aging’s Health Disparities Research Framework [16].

## Materials and methods

### Participants

All participants belong to the Minority Aging Research Study (MARS), an ongoing epidemiologic cohort study of risk factors for cognitive decline and dementia in older African Americans, who begin the study without dementia at baseline. Study recruitment for MARS began in August 2004 and enrollment is ongoing. Prior to recruitment for MARS, study staff engaged in extensive community outreach efforts, such as conducting health fairs and providing presentations on aging-related topics, to foster trust and build aging-related knowledge in African American communities. MARS study staff then moved forward with recruitment efforts by hosting study-related presentations and providing study-specific materials and information to older African Americans in the community to gain interest in study participation. These recruitment activities took place in a variety of community-based settings within and surrounding the greater Chicago metropolitan area, including churches, subsidized senior housing facilities, social clubs and other organizations centering African Americans, as well as social service centers that cater to demographically diverse seniors. Recruitment and retention efforts remain ongoing. Data collection of enrolled participants takes place in individuals’ homes.

Eligibility for MARS participation requires self-identification as African American/Black, at least 65 years of age or older, and no known dementia diagnosis. MARS was not designed to be a representative sample of older African Americans, but rather a convenience sample. MARS participants agree to structured, annual clinical evaluations that include a review of medical history, a comprehensive neuropsychological test battery, and assessment of a wide range of environmental, sociocultural, behavioral, and biological risk factors [17]. A clinician classifies persons regarding dementia using the criteria of the Joint Working Group of The National Institute of Neurological and Communicative Disorders and Stroke and the Alzheimer’s Disease and Related Disorders Association [18]. Only data from the baseline assessment were used for the current study.

Eligibility criteria for the *current* analysis included: 1) self-identification as non-Latino, 2) a non-missing score for perceived stress, and 3) no known dementia diagnosis. Of 752 MARS participants, we excluded 30 people who: 1) identified as Latino (n = 3), 2) had missing values for the measure of perceived stress (n = 1), 3) had not yet completed the clinical evaluation to assess dementia status (n = 7), or 4) met criteria for dementia (n = 19). Thus, the total number eligible for the current analysis was 722 participants. See Table 2 for additional participant descriptive characteristics. MARS was approved by an Institutional Review Board at Rush University Medical Center. All participants provided written informed consent.

**Table 2:** Participant characteristics for all correlates by level.

	Correlate	All Participants (N = 722)	
		Range of Scores	Mean (Standard Deviation) or N (%) [Median (Interquartile Range)^†^]
**Outcome: Perceived Stress**			
	Perceived Stress Scale—Quartile Score ^†^		3.00 (2.00)
**Fundamental Factors**			
	Gender, Men		168 (23%)
	Age	60.2–97.6	73.6 (6.33)
**Level 1: Environmental**			
	Years of Education	0.00–30.00	14.84 (3.48)
	Income	$0 –$75,000+	$30,000
	Life Space ^†^		6.00 (1.00)
	Neighborhood–Safety ^†^		2.00 (1.00)
	Neighborhood–Tension ^†^		1.00 (0.00)
	Neighborhood Violence–Assaulted ^†^		1.00 (1.00)
	Neighborhood Violence–Robbed ^†^		1.00 (1.00)
	Neighborhood Violence–Shot ^†^		1.00 (1.00)
**Level 2: Sociocultural**			
	Financial Burden	2.00–12.00	5.18 (1.66)
	Marital Status, Never Married		30 (4%)
	Marital Status, Married		232 (32%)
	Marital Status, Widowed		268 (37%)
	Marital Status, Divorced		163 (23%)
	Marital Status, Separated		29 (4%)
	Social Network Size	0.00–85.00	6.38 (5.96)
	Social Isolation	1.00–5.00	2.08 (0.61)
	Religion/Frequency of Prayer	1.00–6.00	2.10 (1.05)
**Level 3: Behavioral**			
	Depressive Symptoms ^†^		1.00 (2.00)
	Neuroticism	0.00–43.00	13.88 (6.44)
	John Henryism	4.00–27.00	16.88 (4.89)
	Purpose in Life	2.00–5.00	3.86 (0.45)
	Self-reported Discrimination	0.00–9.00	1.64 (1.98)
	Late Life Cognitive Activity	1.00–4.71	2.93 (0.64)
	Late Life Social Activity	1.00–4.50	2.70 (0.57)
	Physical Activity	0.00–3.00	1.14 (0.93)
	Sleep Quality	0.00–14.00	6.56 (3.05)
**Level 4: Biological**			
	Vascular Risk Factors ^†^		2.00 (1.00)
	Vascular Disease Burden ^†^		0.00 (0.00)
	Body Mass Index	15.98–60.91	30.29 (6.54)
	Global Cognition	-2.09–1.57	0.03 (0.56)
	Memory Complaints	3.00–10.00	7.13 (1.31)

*Note*.

^†^ Median and interquartile range are reported, instead of range of scores and mean (SD), for the following correlates: Perceived Stress Scale—quartile score, life space, safety, tension, violence–assaulted, violence-robbed, violence-shot, depressive symptoms, vascular risk factors, and vascular disease burden.

### Outcome: Perceived stress

Perceived stress was measured using the four-item Perceived Stress Scale [7, 19, 20]. The PSS assesses a person’s subjective evaluation of his or her life as unpredictable, uncontrollable, and overloaded during the past month. The original PSS consisted of 14 items [7] with a modified version consisting of 10 items [19]. The 4-item PSS [19] was developed for research situations requiring brevity in data collection [21] and represents a subset of questions from the 10-item PSS [20]. Participants rated items (e.g., “In the last month, how often have you felt confident in your ability to handle your personal problems?”) along a 5-point Likert scale ranging from 0 (never) to 4 (very often). Two items were reverse-coded, and all scale items were summed to create a total score for each participant. Higher scores indicated higher levels of perceived stress during the past month. Reliability of the 4-item PSS was assessed using an ordinal alpha given the small number of items and the presence of skewness [22]. The ordinal alpha for the 4-item PSS in the current study sample was 0.71, which is considered satisfactory in behavioral research.

Potential corelates of perceived stress were grouped into four levels according to the NIA’s Health Disparities Research Framework [16]. See Table 2 for a modified version of the framework and below for a description of each correlate by level.

### Level 1: Environmental

All participants reported their years of education and income. Income was measured using the Show-Card Method from the Established Populations for Epidemiologic Studies of the Elderly with participants asked to select 1 of 10 levels of total family income [23]. Life space referred to a person’s spatial movement throughout his/her environment and was measured using a modified Life Space Questionnaire [24]. Participants responded “yes” or “no” to six items related to movement in six specific spatial zones (e.g., outside of the bedroom but inside the home is one zone) in the past week. Item responses were summed with higher scores indicating a less constricted or larger life space.

We asked seven questions regarding neighborhood factors. Two questions assessed how safe participants felt in their neighborhood and house using a 5-point Likert scale (1 = very safe to 5 = not safe at all). The two items were: “How safe from crime would you say your neighborhood is?” and “Thinking about the building (house) you live in, how safe from crime would you say it is?” Item responses were reverse-coded and averaged with higher scores indicating more safety. The ordinal alpha for the safety items in the current study sample was 0.72. Two questions assessed neighborhood tension using a 3-point Likert scale (1 = big problem to 3 = not a problem). The two items asked participants to rate tension between people of different racial groups and sexual assault as potential problems in their neighborhoods. Item responses were reverse-coded and averaged with higher scores indicating more tension. The ordinal alpha for the tension items in the current study sample was 0.69. Three questions measured violence (e.g., being assaulted) as a problem in the neighborhood using a 3-point Likert scale (1 = big problem to 3 = not a problem). The three items included: “In the last 12 months, in your neighborhood, have you heard of or do you know about a house which was robbed?” “In the last 12 months, have you heard of or know about a person who was beat up or assaulted?” and “In the last 12 months, have you heard of or know about a person who was shot or killed?” Item responses were reverse-coded and averaged with higher scores indicating more violence.

### Level 2: Sociocultural

Financial burden was measured with four items assessing financial solvency. Three items (e.g., “How much difficulty do you have in meeting the monthly payments on your bills?”) assessed not having enough money for necessities such as food along a 4-point Likert scale (1 = never to 4 = very often). A fourth item assessed the amount of money remaining after covering expenses at the end of the month (1 = money left over, 2 = just enough money left over, and 3 = don’t have enough money to make ends meet). All items were summed with higher values indicating more financial burden. The ordinal alpha for the financial burden measure in the current study sample was 0.88.

We assessed relationship factors including marital status, social network size, and social isolation. For marital status, participants reported their current marital status including never married, married, widowed, divorced, or separated. Social network size was a count of the number of children, family, and friends that a participant saw at least once a month [25]. Social isolation referred to loneliness or feeling remote from others and was measured with the modified Loneliness Scale consisting of five items (e.g., “I experience a general sense of emptiness.”) along a 5-point Likert scale (1 = strongly disagree to 5 = strongly agree) [26]. All items were averaged to create an overall score with higher scores indicating more social isolation. The ordinal alpha for the modified Loneliness Scale in the current study sample was 0.88.

We operationalized religion by asking participants how often they prayed, with response options ranging from 1 (many times a day) to 6 (never or almost never). Scores were reverse coded with higher scores indicating a higher frequency of prayer.

### Level 3: Behavioral

For a marker of affect, we measured depressive symptoms with a modified version of the Center for Epidemiologic Studies—Depression (CES-D) scale [27]. Participants responded “yes” or “no” to 10 items (e.g., “I felt sad.”). Items were summed for a total count with higher scores indicating more depressive symptoms.

The ordinal alpha (using *tetrachoric correlation since the* response variables are *dichotomous)* for the CES-D scale was 0.89.

For personality, we measured neuroticism, or the proneness to experience psychological distress, using 12 items from the NEO Five Factor Inventory [28]. Items (e.g., “I often feel inferior to others.”) were assessed on a 5-point Likert scale (0 = strongly disagree to 4 = strongly agree) with individual items summed to create a total score. Higher scores indicated more neuroticism. We focused on neuroticism due to its documented relationship with stress [10]. The ordinal alpha for the neuroticism measure in the current study sample was 0.82.

We used the John Henryism Scale of Active Coping, a scale specifically developed for African Americans, that reflects a “strong personality predisposition” to cope actively with psychosocial and environmental stressors by expending high levels of effort that may result in negative physiological consequences such as hypertension [29]. We measured John Henryism using nine items (e.g., “Hard work is the best way for a person to get ahead in life.”) along a 4-point Likert scale (0 = not true at all to 3 = completely true). Item responses were summed with higher scores indicating higher levels of John Henryism. The ordinal alpha for the John Henryism Scale of Active Coping in the current study sample was 0.83.

Purpose in life was assessed using a 10-item measure stemming from Ryff’s Scales of Psychological Well-Being [30, 31]. Items measured participants’ ability to derive meaning from life experiences and being goal-directed (e.g., “I enjoy making plans for the future and working them to a reality.”) using a 5-point Likert scale (1 = strongly disagree to 5 = strongly agree). Items were averaged for a total score with higher scores indicating more purpose in life. The ordinal alpha for the purpose in life measure in the current study sample was 0.84.

Self-reported experiences of discrimination referred to a participant’s perception of being treated unfairly in everyday situations [32]. Nine items (e.g., “You are treated with less courtesy than other people.”) were framed in a general context without mention of race, age, or gender and rated along a 4-point Likert scale (1 = often to 4 = never). Responses were recoded to a binary format then summed across items to create a total score (range 0–9), with higher scores indicating more experiences of discrimination [33]. The ordinal alpha for the experiences of discrimination scale in the current study sample was 0.88.

For late-life cognitive activity, participants self-reported their engagement in seven activities (e.g. reading) during the past year using a 5-point Likert scale (1 = every day/almost every day to 5 = once a year or less), as previously described [34, 35]. Scores were reverse-coded and averaged with higher scores indicating more late-life cognitive activity. The ordinal alpha for the late-life cognitive activity measure in the current study sample was 0.65.

Late-life social activity was measured with six items assessing the frequency of participation in events such as visits with family and friends on a 5-point Likert scale (1 = once a year or less to 5 = every day or almost every day) [36]. Items were averaged with higher scores signaling higher levels of late-life social activity. The ordinal alpha for the late-life social activity scale in the current study sample was 0.66.

For physical activity, participants reported if they engaged in three specific activities, including walking for exercise, within the past two weeks. If so, participants reported the number of occasions for each activity.

The ordinal alpha (using tetrachoric correlation since the response variables are dichotomous) for physical activity items was 0.49.

Sleep quality was measured using a modified Pittsburgh Sleep Quality Index (PSQI) [37] and select items from the Berlin Questionnaire [38]. Participants answered 10 questions (e.g., “During the past month, how often did you have trouble falling asleep within 30 minutes?”) representing 6 sleep components (e.g., sleep duration) [39]. Higher scores indicated poorer sleep quality. The ordinal alpha for the sleep quality measure in the current study sample was 0.63.

### Level 4: Biological

Vascular risk factors were the sum of the presence of hypertension, diabetes mellitus, and smoking. Vascular disease burden was the sum of previous or existing myocardial infarction, congestive heart failure, claudication, and stroke. The ordinal alpha (using tetrachoric correlation since the response variables are dichotomous) for vascular risk factors and vascular disease burden was 0.40 and 0.61, respectively. BMI was calculated using weight (kilograms) divided by height (in meters squared).

As previously described, all participants are administered a battery of 21 performance-based cognitive tests that measure a range of abilities [17]. Two tests, the Mini-Mental State Examination (MMSE) and the Complex Ideational Material, were used for descriptive or diagnostic purposes only. The remaining 19 performance-based tests assessed 5 domains of cognitive function (i.e., episodic memory, semantic memory, working memory, perceptual speed, and visuospatial ability). To capitalize on all cognitive data, we used a composite measure of global cognition in analyses, with raw scores on each of the 19 tests converted to z-scores and averaged to yield the composite measure for each participant [35, 40].

We assessed memory complaints using 2 items (“How often do you have trouble remembering things?” and “How does your memory compare to 10 years ago?”) scored along 5-point Likert scales (1 = never to 5 = very often and 1 = much worse to 5 = much better, respectively). Item responses were reverse-coded and summed to create a composite score with higher scores indicating more memory complaints [41]. The ordinal alpha for memory complaints in the current study sample was 0.61.

### Fundamental factors

All participants reported their gender (i.e., male or female) and date of birth to assess age. Participants also reported their ethnicity (e.g., Hispanic: yes or no).

### Analyses

We first examined the distribution of PSS total scores across all participants. PSS total scores were skewed and inflated toward lower values. Due to the distribution of PSS scores in our current study sample, we split PSS total scores into quartiles and transformed the PSS total scores into PSS quartile scores and used the quartile scores in analyses. As described earlier, correlates were grouped into four levels as set forth in the NIA’s Health Disparities Research Framework [16]. For each of the four levels, we performed a separate ordinal logistic regression model assuming proportional odds with PSS quartile scores as the outcome [42]. We used a stepwise approach to select the final model of correlates within each level. The process retained gender and age for all models. For all other correlates, we considered the statistical significance of entering and retention in the model as 0.10 and 0.05, respectively. Finally, each final model within each level was confirmed by repeating the process by using a backward, instead of stepwise, approach [43]. The assumption of proportional odds was established using the score test and was met for all final models (environmental level: *p =* 0.49, sociocultural level: *p =* 0.66, behavioral level: *p =* 0.43, and biological level: *p =* 0.41).

The sample size of 722 participants provided adequate power to detect clinically relevant odds of perceived stress. Calculations for the current study sample size indicated the detection of an effect size of 1.46 and an efficiency probability of 0.93 for the ordinal four-level PSS quartile scores. Power is a function of the distribution of the correlates being tested and was enhanced by the statistical strategy [44, 45]. All analyses were conducted using SAS software, version 9.3 of the SAS system for Linux.

## Results

### Participant characteristics

Participants (N = 722) were non-Latino African Americans and were 77% women, with a mean age of 73.6 years [standard deviation (SD) = 6.33] and had 14.8 (SD = 3.48) mean years of education. Participants had a median MMSE score of 28 (Interquartile Range = 27–29). See Table 1 for additional participant descriptive characteristics. Data can be requested at https://www.radc.rush.edu.

### Statistical models by level

We first grouped correlates into four levels according to the NIA’s Health Disparities Research Framework (i.e., environmental, sociocultural, behavioral, and biological). Then, we performed a separate ordinal logistic regression model with a stepwise approach to select the final model for each level. We confirmed each final model for each level by repeating the process using backward elimination. All models adjusted for gender and age. See Table 3.

**Table 3:** Separate ordinal logistic regression models^†^ for correlates grouped into four levels; with main effects for each correlate while controlling for other correlates within each level; perceived stress scale–quartile score is the outcome.

	Correlate	Odds Ratio	95% Confidence Interval	Standard Error	*p*-Value
**Level 1: Environmental**					
	Years of Education	1.03	0.99–1.08	0.02	0.17
	Income	0.91	0.85–0.97	0.03	0.003
	Life Space	0.70	0.59–0.83	0.09	< 0.001
	Neighborhood–Safety	0.64	0.51–0.79	0.11	< 0.001
	Neighborhood–Tension	1.93	1.20–3.10	0.24	0.006
	Neighborhood Violence–Assaulted	1.09	0.82–1.43	0.14	0.55
	Neighborhood Violence–Robbed	0.90	0.71–1.14	0.12	0.37
	Neighborhood Violence–Shot	0.83	0.64–1.08	0.13	0.16
**Level 2: Sociocultural**					
	Financial Burden	1.52	1.38–1.67	0.05	< 0.001
	Marital Status	0.89	0.77–1.04	0.08	0.15
	Social Network Size	0.96	0.93–0.99	0.01	0.005
	Social Isolation	1.95	1.51–2.51	0.13	< 0.001
	Religion/ Frequency of Prayer	1.05	0.91–1.20	0.07	0.51
**Level 3: Behavioral**	
	Depressive Symptoms	1.29	1.07–1.56	0.10	0.009
	Neuroticism	1.05	1.00–1.11	0.03	0.06
	John Henryism	0.95	0.90–1.00	0.03	0.07
	Purpose in Life	0.49	0.24–1.00	0.36	0.05
	Perceived Discrimination	1.08	0.93–1.25	0.08	0.32
	Late Life Cognitive Activity	0.88	0.58–1.35	0.22	0.57
	Late Life Social Activity	0.93	0.55–1.58	0.27	0.80
	Physical Activity	0.83	0.64–1.10	0.14	0.19
	Sleep Quality	1.04	0.95–1.14	0.05	0.39
**Level 4: Biological**					
	Vascular Risk Factors	0.87	0.74–1.02	0.08	0.10
	Vascular Disease Burden	1.14	0.90–1.44	0.12	0.30
	Body Mass Index	1.04	1.02–1.06	0.01	< 0.001
	Global Cognition	0.75	0.57–0.97	0.14	0.03
	Memory Complaints	1.12	1.01–1.24	0.05	0.03

*Note*.

^†^All models included terms for gender and age.

#### Level 1: Environmental

Correlates included in the environmental level were years of education, income, life space, neighborhood safety, neighborhood tension, and neighborhood violence. A range of correlations existed among environmental level factors (r_s_ = <0.41), supporting their inclusion within this level. Higher income, larger life space, and feeling more safe in one’s neighborhood were protective factors against having greater levels of perceived stress. For example, an increase in 1 point in life space was associated with 30% lower odds of having higher perceived stress. In contrast, feeling more tension in one’s neighborhood was a risk factor for having greater levels of perceived stress. Neither years of education nor experiencing violence in one’s neighborhood were associated with perceived stress.

#### Level 2: Sociocultural

Correlates included in the sociocultural level were financial burden, marital status, social network size, social isolation, and religion/ frequency of prayer. A range of correlations existed among sociocultural level factors (r_s_ = <0.23), supporting their inclusion within this level. A larger social network size was a protective factor against having greater levels of perceived stress. For example, an increase of 1 person in social network size was associated with 4% lower odds of having higher perceived stress. More financial burden and more social isolation were risk factors for having greater levels of perceived stress. Marital status and prayer frequency did not have a relationship with perceived stress.

#### Level 3: Behavioral

Correlates in the behavioral level were depressive symptoms, neuroticism, John Henryism, purpose in life, self-reported discrimination, late-life cognitive activity, late-life social activity, physical activity, and sleep quality. A range of correlations existed among behavioral level factors (r_s_ = <0.46), supporting their inclusion within this level. More purpose in life was a protective factor against having greater levels of perceived stress, with an increase of 1 SD of purpose in life (0.45) being associated with 27% lower odds of having higher perceived stress. More depressive symptoms were a risk factor for having greater levels of perceived stress. An increase of 1 depressive symptom was associated with 44% higher odds of having higher perceived stress. Neither neuroticism, John Henryism, self-reported discrimination, late-life cognitive activity, late-life social activity, physical activity, nor sleep quality had a relationship with perceived stress.

#### Level 4: Biological

Correlates included in the biological level were vascular risk factors, vascular disease burden, BMI, global cognition, and memory complaints. A range of correlations existed among biological level factors (r_s_ = <0.24), supporting their inclusion within this level. Higher global cognition was a protective factor against having greater levels of perceived stress. Higher BMI and more memory complaints were risk factors for having greater levels of perceived stress. Neither vascular risk factors nor vascular disease burden had a relationship with perceived stress.

## Discussion

The purpose of this study was to identify correlates of perceived stress among community-dwelling, older African Americans. Higher levels of perceived stress have been linked to poorer health outcomes across the life course, but correlates of perceived stress among older adults, especially older racial minorities, remain less understood. Perceived stress is a potentially modifiable factor with a documented role in health outcomes in older adults [10, 11]. Hence, understanding correlates associated with perceived stress in older African Americans may facilitate the development and implementation of educational/informational tools and intervention strategies aimed toward stress reduction in this vulnerable population. Correlates represented one of four levels set forth by the NIA’s Health Disparities Research Framework [16]: 1) environmental, 2) sociocultural, 3) behavioral, and 4) biological. Consistent with our hypotheses, we found perceived stress associated with correlates from each of the four levels, supporting the idea that a multi-level analysis approach is important in understanding aging and relevant outcomes in diverse older adults. Some correlates were associated with lower levels of perceived stress, while others were associated with higher levels of perceived stress. Within the same level, correlates operated differently in their relationship with perceived stress. Specifically, within the environmental level, lower levels of perceived stress were associated with having a higher income, a larger life space, and feeling safer in one’s neighborhood. Within the sociocultural level, lower levels of perceived stress were associated with having a larger social network. Within the behavioral level, lower levels of perceived stress were associated with having more purpose in life. Within the biological level, lower levels of perceived stress were associated with having higher global cognition. Conversely, higher levels of perceived stress were associated with feeling more tension in one’s neighborhood (environmental level); having more financial burden and more social isolation (sociocultural level); having more depressive symptoms (behavioral level); and having a higher BMI and more memory complaints (biological level).

We add to the relatively small body of research focused on perceived stress as an outcome among older adults, especially older racial minorities. Previous research has suggested that environmental, sociocultural, and behavioral factors are associated with perceived stress among older adults, but studies have not specifically examined older African Americans. One previous study found that a larger social support network was associated with lower levels of perceived stress among older adults [46], similar to current study findings. Furthermore and similar to current study findings, previous research has suggested that negative neighborhood-related factors, more financial burden, and more social isolation are associated with higher levels of perceived stress in older adults [12, 47, 48]. In the current study, unsurprisingly, higher income was associated with lower levels of perceived stress. We also found a relationship between more depressive symptoms and higher levels of perceived stress. Depressive symptoms, common among older adults, including among older racial minorities [49], have been postulated to have a confounding and bidirectional relationship with perceived stress across the life course [50]. However, due to our cross-sectional design, we were unable to examine the directionality of the effect.

Current study findings also demonstrate novel correlates associated with perceived stress among older African Americans. Among environmental factors in the current study, a larger life space was associated with lower levels of perceived stress. Larger life space has been linked to positive factors in aging including better cognitive function, more purpose in life, greater mobility, and more social involvement among older adults, including older African Americans [24, 51, 52]. Similarly, among behavioral factors in the current study, more purpose in life was associated with lower levels of perceived stress. More purpose in life has been associated with decreased mortality risk in middle- to older- aged adults [53, 54], certainly a finding that is consistent with lower levels of perceived stress. Current study findings also suggest that correlates at the biological level are associated with perceived stress, such as global cognition and memory complaints. Consistent with current study findings, higher levels of perceived stress have been linked to faster rates of cognitive decline in MARS as well as in a population-based study of older African Americans and Whites [10, 11], and more memory complaints [55] among older adults. However, these previous studies all included perceived stress as a predictor, not as an outcome as in our current study, but nevertheless supports the important role of cognitive health for perceived stress among older African Americans. Lastly, we found a relationship between higher BMI and higher levels of perceived stress, but the basis of the association in older African Americans remains unclear. Higher BMI has been linked to poorer self-rated health in a sample of middle-to-older aged African Americans [56]. It is possible that higher BMI is associated with negative perceptions of health among older African Americans and this functions as a stressor, but more research is needed to understand the directionality and underlying mechanisms of this association.

Overall, the current study takes an important step toward identifying and understanding antecedents of perceived stress among older African Americans—a specific subgroup of older adults who may be at risk for experiencing high levels of perceived stress and, arguably, experiencing poorer health outcomes in aging linked to higher levels of exposure to chronic stress. Correlates associated with perceived stress among older African Americans were categorized using a published framework [16] that considers four interrelated levels of factors–environmental, sociocultural, behavioral, and biological–associated with health disparities in aging. These correlates represented both positive and negative factors within each level, including factors that have been traditionally considered in studies of perceived stress with diverse populations (e.g., income and cognition) and novel correlates such as purpose in life and neighborhood conditions. Furthermore, most correlates are potentially modifiable (e.g., social network size and social isolation). While it may not be possible to reduce someone’s perceptions of stress, by focusing on these modifiable factors, researchers and practitioners can develop and deliver both effective and culturally compatible educational tools and intervention strategies geared toward addressing and coping with perceptions of stress among older African Americans. Conversely, some correlates are not modifiable, primarily financial (i.e., income and financial burden) and neighborhood factors (i.e., safety and tension) associated with perceived stress. It is possible that these non-modifiable factors “mask” structural racism or other intertwined inequities commonly faced by African Americans across the lifespan. It remains critical for researchers and practitioners to partner with policy makers to establish and implement structural changes needed to address these non-modifiable correlates or sources of higher levels of perceived stress. Structural strategies focused on, for example, income parity and housing equity for African Americans across the lifespan may decrease or buffer higher levels of perceived stress that are resistant to related educational resources and intervention strategies. Ultimately, future research is needed to examine antecedents of perceived stress among older African Americans to further identify modifiable and non-modifiable factors that may mitigate or prevent higher levels of perceived stress and lead to healthier aging for this population.

### Strengths and limitations

This study has limitations. First, participants came from a volunteer cohort study in the Midwest and tended to be healthier and have more years of education than typical older African Americans. Hence, current study participants may have overall lower levels of perceived stress and findings may not be generalizable to older African Americans across the United States. The study should be replicated in a population-based sample. Second, we used the four-item PSS to measure perceived stress. Although the 4-item version has been validated in previous studies, including with older adults, the 10-item and 14-item versions may have yielded different results. Lastly, due to our cross-sectional design, we cannot infer causality and it is not possible to determine whether our correlates predict perceived stress, perceived stress predicts correlates, or both. Future research is needed to explore predictive factors of perceived stress using longitudinal designs. This study also has strengths including a well-characterized cohort of older African Americans, data on a wide range of theoretically- and literature-driven correlates of perceived stress, and use of the NIA’s Health Disparities Research Framework [16] that grounded our categorization of correlates into levels of influence for perceived stress.
